# The importance of Structural and Functional Analysis of Extracts in Plants

**DOI:** 10.3390/plants10061225

**Published:** 2021-06-16

**Authors:** Stefania Lamponi

**Affiliations:** Department of Biotechnologies, Chemistry and Pharmacy and SienabioACTIVE s.r.l., University of Siena, Via Aldo Moro 2, 53100 Siena, Italy; stefania.lamponi@unisi.it; Tel.: +39-0577-234386; Fax: +39-0577-234254

## 1. Introduction

Plants and their extracts have traditionally been used against various pathologies and in some regions are the only therapeutic source for the treatment and prevention of many chronic diseases [1]. Their numerous beneficial effects on human health are due to their content of natural bioactive compounds, molecules capable of modulating metabolic processes with positive properties such as antioxidant effects, inhibition of receptor activities and enzymes and induction of gene expression [2].

Many factors, such as agronomical and processing conditions used in plant cultivation, extraction methods and solvents, may influence the chemical profile of the herbal preparations and, consequently, their pharmacological activities [3,4,5]. For these reasons, chemical analysis should be always performed on each plant extract in order to correlate the type and the amount of phytochemicals present with its bioactivity so as to better select its specific applications [6].

Although numerous advances have been made in recent years regarding the structural and functional analysis of extracts in plants, this topic deserves more attention from the academic community; therefore, we have edited two Special Issues that include numerous research articles and reviews reflecting the advancements made thus far in this field.

## 2. Plant Extracts’ Phytochemicals and Their Bioactivity

The health benefits of plant extracts mainly depend on their secondary metabolites, i.e., substances produced by plants that make them competitive in their own environment [7]. Secondary metabolites vary widely in chemical structure (types of functional groups, number and position with respect to the carbon skeleton, substitution in the aromatic ring, stereochemistry, side chain length, saturation, etc.) [8] and the most extensively studied are those with antioxidant properties that protect cellular systems from oxidative damage through a variety of mechanisms able to reduce the risk of chronic diseases such as cancer and cardiovascular disease [9].

The most important classes of secondary metabolites in plants and in their extracts are alkaloids, phytoestrogens, carotenoids, tocopherols, terpenes and phenolics.

Alkaloids are plant-derived compounds containing one or more nitrogen atoms, usually in a heterocyclic ring (amine functional group). They derive from amino acids as well as proteins, from which they differ in being alkaline [7]. Alkaloids demonstrate a large spectrum of activities and among them, there are compounds showing antibacterial, antiviral, anti-inflammatory and anticancer properties. For example, *Dicentra spectabilis*, *Corydalis lutea*, *Mahonia aquifolia*, *Fumaria officinalis*, *Meconopsis cambrica* and *Macleaya cordata* plant extracts are cytotoxic against human squamous carcinoma and adenocarcinoma cells and the extracts obtained from the stem bark of *Rutidea parviflora* against ovarian cancer [10]. The bisbenzylisoquinoline alkaloid, named curine, is able to modulate inflammatory effects in mice, by inhibiting macrophage activation, production of cytokines and neutrophil recruitment, and decreasing nitric oxide levels [11]. The antibacterial activity of alkaloids has been described for nigritanine, an alkaloid obtained from *Strychnos nigritana* belonging to the family of Loganiaceae, against *Staphylococcus aureus*, one of the most important pathogenic bacteria diffused worldwide [12]. Moreover, extracts from *Lepidium meyenii*, a plant in the Brassicaceae family rich in alkaloids, shows a strong antioxidant effect, higher than that of phenols [13]. Many alkaloids act on the nervous system. For example, poppy is narcotic, caffeine and nicotine are stimulants, while cocaine is an anesthetic, and scopolamine induces “twilight sleep”. Codeine is frequently used in medical practice to suppress severe coughing [7].

Phytoestrogens are polyphenolic and non-steroid compounds that have a similar structure and biological activity as human estrogens. They are divided into two main subgroups, isoflavonoids and lignans. Isoflavones are divided into isoflavones and cumestanes, and the most representative compound of this second subgroup is coumestrol. Lignans include matairesinol, secoisolariciresinol, lariciresinol, pinoresinol and their metabolites, enterodiol, enterolactone and equol. Numerous studies reported in the literature have shown that phytoestrogens can have protective effects in estrogen-dependent diseases. This bioactivity is due to their structural and/or functional similarity to estradiol and to their capacity to bind the human estrogen receptors. Furthermore, the use of phytoestrogens can have a positive effect on insomnia and cognitive function in neuronal pathologies such as Alzheimer’s disease. Phytoestrogens also exhibit antioxidant activity by acting as scavengers of free radicals or forming chelating complexes with metal ions [14,15].

Carotenoids are natural pigments and one of the main classes of phytochemicals in plants. They are derived from acyclic C_40_ isoprenoid lycopene, which can be classified as a tetraterpene [16]. Carotenoids are divided into carotenes (i.e., α-carotene, β-carotene, lycopene) and xanthophylls, which represent the oxygenated carotenoids fraction (lutein, zeaxanthin and β-cryptoxanthin). The importance of carotenoids is correlated, over their role as precursors of vitamin A (α-carotene, β-carotene and β-cryptoxanthin), with numerous bioactivities. In fact, it has been shown that carotenoids have antioxidant and antitumor activity, regulate gene function and gap-junction communication and modulate the immune response [17,18,19]. Carotenoids may protect light-exposed tissues from photo-oxidative damage which could be involved in the pathobiochemistry of several diseases affecting the skin and the eye. Lutein and zeaxanthin are the predominant carotenoids of the retina and are considered to act as photoprotectants, preventing retinal degeneration. Moreover, β-carotene is also used as an oral sun protectant for the prevention of sunburn and has been shown to be effective either alone or in combination with other carotenoids or antioxidant vitamins [20].

Tocopherols, together with carotenoids, are the most abundant group of lipid-soluble antioxidants in chloroplasts [21]. α-, β-, δ- and γ-tocopherols and α-, β-, δ- and γ-tocotrienols are different forms of vitamin E and, among them, α-tocopherol is the most predominant and active form in human tissues. Tocopherols are antioxidants, free-radical scavengers and membrane stabilizers, protect thylakoid components from oxidative damage, are involved in electron transport reactions, in the prevention of light-induced pathologies of skin and eyes and in photophosphorylation and also have hypocholestemic health benefits [22,23,24]. Other bioactivities of tocopherols, not correlated with their antioxidant effects, are inhibition of platelet aggregation and monocyte adhesion and anti-proliferative and neuroprotective effects [25].

Terpenes are hydrocarbons based on combinations of dimethylallyl pyrophosphate and isoprenyl diphosphate/pyrophosphate, while terpenoids (also known as isoprenoids) are terpenes with an oxygen moiety and additional structural rearrangements. Terpenoids are classified on the basis of the number of carbon atoms present in their structure in hemiterpenoids (C_5_), monoterpenoids (C_10_), homoterpenoids (C_11,16_), sesquiterpenoids (C_15_), diterpenoids (C_20_), sesterpenoids (C_25_), triterpenoids (C_30_), tetraterpenoids (C_40_) and polyterpenoids (C_>40_, higher-order terpenoids) [26]. Recent studies have shown that many triterpenoids are effective and have pharmacological activities against cancer and other pathologies, such as cardiovascular diseases, diabetes and neurological disorders [27]. The pharmacological properties of triterpenoids in cancer prevention are attributed to multiple mechanisms, including antioxidant, anti-inflammatory and cell cycle regulatory properties, as well as epigenetic/epigenomic regulation.

Phenolic compounds are a class of molecules whose basic structural feature is an aromatic ring of hydroxyl groups. They include flavonoids (i.e., flavonols, flavones, flavan-3-ols, anthocyanidins, flavanones, isoflavones, condensed tannins) and nonflavonoids (i.e., phenolic acids, hydroxycinnamates, stilbenes, hydrolyzable tannins) depending on the number and arrangement of their carbon atoms. These compounds have high antioxidant activity, a protective effect against chronic pathologies such as cancer, inflammatory diseases and bacterial disorders and favorable effects in reducing the risks of coronary heart disease [28]. Much epidemiological evidence also reports their ability to reduce diabetes and human neurodegenerative pathologies, such as Parkinson’s and Alzheimer’s diseases. Moreover, anti-analgesic, anti-allergic, cardioprotective and anti-diabetic activities have also been documented for food phenolics [28].

## 3. Future Perspectives

All the studies reported in the literature demonstrated that natural plant products are an abundant source of biologically active compounds, many of which can be considered as the basis for the development of new lead chemicals for pharmaceuticals. Although numerous advances have been made in recent years in this field, further research is needed in order to translate experimental in vitro results into clinical applications. In particular, studying the in vivo mechanisms, identifying epigenetic regulatory switches, finding new analogs and increasing the bioavailability of plants metabolites, could help to identify more effective compounds to prevent and/or to treat chronic diseases. For the selection of natural compounds intended to represent the next generation of therapies based on natural formulations, and to enable them to compete with modern drugs, it is necessary to investigate different aspects of the processes necessary to obtain them, such as extraction techniques, and evaluation of the quality and of the bioactivity of crude extracts and their combinations. Moreover, new and advanced techniques for their purification and efficient animal studies along with appropriate clinical trials are required for justified use of these plant extracts with safety and efficacy.

## References

[1] Passero L.F., Laurenti M.D., Santos-Gomes G., Soares Campos B.L., Sartorelli P., Lago J.H. (2014). Plants used in traditional medicine: Extracts and secondary metabolites exhibiting antileishmanial activity. Curr. Clin. Pharmacol..

[2] Carbonell-Capella J.M., Barba F.J., Esteve M.J., Frígola A. (2014). Quality parameters, bioactive compounds and their correlation with antioxidant capacity of commercial fruit-based baby foods. Food Sci. Technol. Int..

[3] Ribeiro A., Caleja C., Barros L., Santos-Buelga C., Barreiro M.F., Ferreira I.C.F.R. (2016). Rosemary extracts in functional foods: Extraction, chemical characterization and incorporation of free and microencapsulated forms in cottage cheese. Food Funct..

[4] Mulinacci N., Innocenti M., Bellumori M., Giaccherini C., Martini V., Michelozzi M. (2011). Storage method, drying processes and extraction procedures strongly affect the phenolic fraction of rosemary leaves: An HPLC/DAD/MS study. Talanta.

[5] Bicchi C., Binello A., Rubiolo P. (2000). Determination of phenolic diterpene antioxidants in rosemary (*Rosmarinus officinalis* L.) with different methods of extraction and analysis. Phytochem. Anal..

[6] Lamponi S., Baratto M.C., Miraldi E., Baini G., Biagi M. (2021). Chemical Profile, Antioxidant, Anti-Proliferative, Anticoagulant and Mutagenic Effects of a Hydroalcoholic Extract of Tuscan Rosmarinus officinalis. Plants.

[7] Teoh E.S. (2016). Secondary Metabolites of Plants. Medicinal Orchids of Asia.

[8] Segneanu A.E., Velciov S.M., Olariu S., Cziple F., Damian D., Grozescu I., Asao T. (2017). Bioactive Molecules Profile from Natural Compounds. Amino Acid—New Insights and Roles in Plant and Animal.

[9] Kris-Etherton P.M., Hecker K.D., Bonanome A., Coval S.M., Binkoski A.E., Hilpert K.F., Griel A.E., Etherton T.D. (2002). Bioactive compounds in foods: Their role in the prevention of cardiovascular disease and cancer. Am. J. Med..

[10] Lelario F., De Maria S., Rivelli A.R., Russo D., Milella L., Bufo S.A., Scrano L. (2019). A Complete Survey of Glycoalkaloids Using LC-FTICR-MS and IRMPD in a Commercial Variety and a Local Landrace of Eggplant (*Solanum melongena* L.) and their Anticholinesterase and Antioxidant Activities. Toxins.

[11] Ribeiro-Filho J., Carvalho Leite F., Surrage Calheiros A., de Brito Carneiro A., Alves Azeredo J., Fernandes de Assis E., da Silva Dias C., Regina Piuvezam M.T., Bozza P. (2019). Curine Inhibits Macrophage Activation and Neutrophil Recruitment in a Mouse Model of Lipopolysaccharide-Induced Inflammation. Toxins.

[12] Casciaro B., Calcaterra A., Cappiello F., Mori M., Loffredo M.R., Ghirga F., Mangoni M.L., Botta B., Quaglio D. (2019). Nigritanine as a New Potential Antimicrobial Alkaloid for the Treatment of Staphylococcus aureus-Induced Infections. Toxins.

[13] Gan J., Feng Y., He Z., Li X., Zhang H. (2017). Correlations between Antioxidant Activity and Alkaloids and Phenols of Maca (*Lepidium meyenii*). J. Food Qual..

[14] Gajić I., Savic Gajic I.M., Tačić A., Savic I.M. (2017). Classification and biological activity of phytoestrogens: A review. Adv. Technol..

[15] Martins S., Cristóbal N., Aguilar J.A., Teixeira S.I. (2012). Mussatto, Bioactive compounds (phytoestrogens) recovery from Larrea tridentata leaves by solvents extraction. Sep. Purif. Technol..

[16] Arvayo-Enríquez H., Mondaca-Fernández I., Gortárez-Moroyoqui P., López-Cervantes J., Rodríguez-Ramírez R. (2013). Carotenoids extraction and quantification: A review. Anal. Methods.

[17] Adadi P., Barakova N.V., Krivoshapkina E.F. (2018). Selected Methods of Extracting Carotenoids, Characterization, and Health Concerns: A Review. J. Agric. Food Chem..

[18] Rao A.V., Rao L.G. (2007). Carotenoids and human health. Pharmacol. Res..

[19] Paradiso V.M., Castellino M., Renna M., Santamaria P., Caponio F. (2020). Setup of an Extraction Method for the Analysis of Carotenoids in Microgreens. Foods.

[20] Stahl W., Sies H. (2005). Bioactivity and protective effects of natural carotenoids. Biochim. Biophys. Acta.

[21] Della Penna D., Pogson B.J. (2006). Vitamin synthesis in plants: Tocopherols and carotenoids. Annu. Rev. Plant. Biol..

[22] Fryer M.J. (1992). The antioxidant effects of thylakoid vitamin E (α-tocopherol). Plant. Cell Environ..

[23] Giasuddin A.S.M., Diplock A.T. (1981). The influence of vitamin E in membrane lipids of mouse fibroblasts in culture. Arch. Biochem. Biophy..

[24] Qureshi A.A., Bradlow B.A., Brace L., Manganello J., Peterson D.M., Pearce B.C., Wright J.J.K., Grapor A., Elson C.E. (1995). Response of hypercholesterolemic subjects to administration of tocotrienols. Lipids.

[25] Rimbach G., Minihane A.M., Majewicz J., Fischer A., Pallauf J., Virgli F., Weinberg P.D. (2002). Regulation of cell signaling by vitamin E. Proc. Nutr. Soc..

[26] Boncan D.A.T., Tsang S.S.K., Li C., Lee I.H.T., Lam H.-M., Chan T.F., Hui J.H.L. (2020). Terpenes and Terpenoids in Plants: Interactions with Environment and Insects. Int. J. Mol. Sci..

[27] Li S., Kuo H.D., Yin R., Wu R., Liu X., Wang L., Hudlikar R., Peter R.M., Kong A.N. (2020). Epigenetics/epigenomics of triterpenoids in cancer prevention and in health. Biochem. Pharmacol..

[28] Shahidi F., Yeo J. (2018). Bioactivities of Phenolics by Focusing on Suppression of Chronic Diseases: A Review. Int. J. Mol. Sci..

